# Evaluation of prophylactic dosages of Enoxaparin in non-surgical elderly patients with renal impairment

**DOI:** 10.1186/s40360-019-0308-8

**Published:** 2019-05-07

**Authors:** Nibal Chamoun, Hady Ghanem, Ahmad Hachem, Essa Hariri, Christelle Lteif, Hanine Mansour, Hani Dimassi, Richard Zalloum, Georges Ghanem

**Affiliations:** 1 Department of Pharmacy Practice, School of Pharmacy, Lebanese American University, PO BOX 36, Byblos, Lebanon; 2 Hematology Oncology Division, Lebanese American University Medical Center Rizk Hospital, Beirut, Lebanon; 3 Pediatrics Division, American University of Beirut Medical Center, Riad El Solh, Beirut, Lebanon; 4 Division of Cardiology, University of Massachusetts Medical School, Worcester, MA USA; 5 Department of Pharmaceutical Sciences, School of Pharmacy, Lebanese American University, Byblos, Lebanon; 6 Cardiology Division, Lebanese American University Medical Center Rizk Hospital, Beirut, Lebanon

**Keywords:** Thromboprophylaxis, Enoxaparin, Renal impairment, Elderly, Anti-Xa, Venous thromboembolism

## Abstract

**Background:**

Thromboprophylaxis dosing strategies using enoxaparin in elderly patients with renal disease are limited, while dose adjustments or monitoring of anti-Xa levels are recommended. We sought to evaluate the efficacy and safety of enoxaparin 20 mg versus 30 mg subcutaneously daily by comparing anti-Xa levels, thrombosis and bleeding.

**Methods:**

We conducted a prospective, single-blinded, single-center randomized clinical trial including non-surgical patients, 70 years of age or older, with renal disease requiring thromboprophylaxis. Patients were randomized to receive either 20 mg or 30 mg of enoxaparin. The primary endpoint was peak anti-Xa levels on day 3. Secondary endpoints included trough anti-Xa levels on day 3, achievement of within range prophylactic target peak anti-Xa levels and the occurrence of hemorrhage, thrombosis, thrombocytopenia or hyperkalemia during hospitalization.

**Results:**

Thirty-two patients were recruited and sixteen patients were randomized to each arm. Mean peak anti-Xa level was significantly higher in 30 mg arm (*n* = 13) compared to the 20 mg arm (*n* = 11) 0.26 ± 0.11, 95%CI (0.18–0.34), versus 0.14 ± 0.09, 95CI (0.08–0.19) UI/ml, respectively; *p* = 0.004. Mean trough anti-Xa level was higher in 30 mg arm (*n* = 10) compared to the 20 mg arm (*n* = 16), 0.06 ± 0.03, 95CI (0.04–0.08) versus 0.03 ± 0.03, 95CI (0.01–0.05) UI/ml, respectively; *p* = 0.044. Bleeding events reported in the 30 mg arm were one retroperitoneal bleed requiring multiple transfusions, and in the 20 mg arm one hematuria. No thrombotic events were reported.

**Conclusion:**

Peak anti-Xa levels provided by enoxaparin 20 mg were lower than the desired range for thromboprophylaxis in comparison to enoxaparin 30 mg.

**Trial registration:**

The trial was retrospectively registered on ClinicalTrials.gov identifier: NCT03158792. Registered: May 18, 2017.

## Introduction

Venous thromboembolism (VTE) is a common and preventable cause of hospital-related morbidity and mortality [1, 2]. While thromboprophylaxis dosing strategies with low molecular weight heparins (LMWHs) are well characterized for patients with normal renal function, they are less established in renal impairment due to limited published literature, as these patients were excluded from several landmark clinical trials [3–8]. Dosage adjustment or monitoring of prophylactic doses of LMWHs is recommended in select clinical scenarios, such as renal impairment, by using the chromogenic assay anti-Xa [9–12]. It is also important to note that patients with renal impairment are at an increased risk of thrombosis and bleeding [13]. Moreover, elderly patients with a concomitant picture of renal impairment are also underrepresented in clinical trials thus; thromboprophylaxis presents a challenging situation for these patients [14].

A gap in the literature exists regarding efficacy and safety of different prophylactic doses of LMWHs in severe renal impairment with creatinine clearance (CrCl) less than 30 mL/min [9, 15]. Prophylactic doses of tinzaparin and dalteparin seem to be safe in renal impairment, whereas drug accumulation has been demonstrated with enoxaparin [3, 11, 16, 17]. Moreover, an inverse relationship has been demonstrated via pharmacokinetic studies between CrCl and LMWH anti-Xa levels, especially with enoxaparin, in patients with severe renal impairment [3, 5, 9, 18]. Specifically, studies in elderly patients with renal dysfunction receiving prophylactic enoxaparin 40 mg resulted in elevated anti-Xa levels, especially in those with severe renal dysfunction [4, 5, 19]. Despite that enoxaparin has shown to accumulate in elderly patients with renal impairment, it is still widely used and has been studied in medically ill elderly patients [14, 17, 20].

To date, there is no clear recommendation for the appropriate thromboprophylaxis dosing using enoxaparin among elderly patients with renal impairment, and unfractionated heparin is still preferred over LMWH in those patients [21]. Although heparin is preferred, its use has associated with a higher risk of bleeding in comparison to LMWH [22]. Moreover physicians and nurses may also prefer LMWH over heparin because of the less frequent administration. Although enoxaparin is commonly prescribed, manufactures of enoxaparin do not have a unified recommendation for dose adjustment in renal impairment. Doses of 20 mg or 30 mg subcutaneously (SC) once daily are both used, depending on the country it’s being used in [23–25]. Prescribers usually adopt institution-specific strategies or opt to prescribe the dose that is available as a prefilled syringe. In Lebanon, 20 mg of enoxaparin is available as a prefilled syringe and hence many providers select this dosing strategy. A recent retrospective study in Lebanon showed that enoxaparin 20 mg as thromboprophylaxis in renal impairment resulted in acceptable rates of thromboembolism and bleeding [26].

To the best of our knowledge, there is no published data that compares the current recommended VTE prophylactic dosages of enoxaparin 20 mg versus 30 mg SC in non-surgical patients with renal impairment, CrCl < 30 ml/min. Therefore, this study aims to evaluate the efficacy and safety between two different recommended dosing strategies of enoxaparin, 20 mg versus 30 mg subcutaneously daily for VTE prophylaxis among elderly patients with a CrCl ≤35 ml/min, by comparing anti-Xa levels, thrombosis and bleeding.

We hypothesize that doses of enoxaparin 20 mg versus 30 mg subcutaneous in elderly patients with renal impairment may achieve different levels anti-Xa levels thus possibly affecting the efficacy of thromboprophylaxis in this setting.

## Methods

### Trial design

We conducted a prospective, single-blinded, single-center, randomized clinical trial (ClinicalTrials.gov identifier: NCT03158792) at the Lebanese American University Medical Center – Rizk Hospital in Beirut, Lebanon.

### Participants, interventions, and study outcomes

Between October 2015 and July 2017, 32 elderly patients from both acute and critical care settings were enrolled by all study investigators during medical rounds. The inclusion and exclusion criteria of this trial were based on previous publications [4, 27]. The trial included non-surgical patients, 70 years of age or older, with renal impairment, defined by CrCl ≤35 ml/min based on the Cockcroft-Gault formula, and with an indication for pharmacological VTE prophylaxis according to the optional hospital risk assessment form or at the discretion of the physician [28]. To calculate the CrCl, the actual body was used if actual body weight was less than ideal body weight. If actual body weight was greater than ideal body weight (IBW) by more than 20%, adjusted body weight was used. Adjusted body weight = IBW + 0.4 (actual body weight - IBW). The renal impairment cutoff was defined as a CrCl ≤35 ml/min instead of a CrCl < 30 ml/min because of institution specific practices in dosing anticoagulants to elderly patients with renal impairment. We excluded patients with an indication for a therapeutic dose of anticoagulant treatment; knee surgery or hip surgery within 10 to 35 days, respectively; recent surgery, hemodialysis, peritoneal dialysis, trauma or bleeding; history of heparin-induced thrombocytopenia; known hypersensitivity to enoxaparin; an excessive risk of bleeding and not eligible for pharmacological thromboprophylaxis based on physician assessment or due to any of the 3 major risk factors including active gastroduodenal ulcer, bleeding within the past three months prior to hospital admission, or a platelet count of < 50,000 platelets per microliter. Although obesity, defined as BMI ≥30 kg/m2, was not in the initial set of the exclusion criteria, we excluded patients with obesity in order not to bias the anti-Xa levels or undertreat patients due to specific clinical considerations while dosing thromboprophylaxis in extreme body weight [29–31]. We assigned patients into one of 2 arms: enoxaparin 20 mg or 30 mg SC daily. The primary endpoint was the serum peak anti-Xa levels, measured on day 3 of thromboprophylaxis, 4 h after the third enoxaparin dose. Secondary endpoints included trough anti-Xa levels on day 3, measured before the third enoxaparin dose, the number of patients achieving a prophylactic target peak anti-Xa levels within range defined as 0.2–0.4 IU/ml based on expert opinion, and the occurrence of hemorrhage or VTE within 30 days assessed from randomization till the date of hemorrhage or VTE or the date of discharge, whichever came first [15, 32]. Hemorrhage was defined according to the GUSTO criteria [33]. VTE was defined as objectively detected deep venous thrombosis (DVT) or pulmonary embolism (PE) by duplex ultrasonography or contrast enhanced computed tomography scan, respectively. Other secondary endpoints included the occurrence of thrombocytopenia defined as a platelet count of less than 150,000 per microliter and hyperkalemia, defined as a potassium level above 4.7 mEq/L as per hospital laboratory limits. Since both doses are approved for VTE prophylaxis, no dose adjustments were recommended based on the anti-Xa levels. Clinical endpoints were not selected as the primary endpoints due to the limitation in the expected time to recruit a high number of patients to power the clinical endpoint. This study was approved by the Lebanese American University Institutional Review Board (LAU IRB), approval number is LAU.SOP.NC1.25/Jun/2015 and performed in accordance with the ethical standards as laid down in the 1964 Declaration of Helsinki and its later amendments or comparable ethical standards. Informed consent was obtained from all study subjects or their legal authorized representative in compliance with ethics committee regulations. Given that thromboprophylaxis in non-surgical patients has been proven to benefit patients with no increase in harm, if patients withdrew consent, they were not included within the study but continued to receive thromboprophylaxis at the discretion of their physician.

### Sample size, randomization, blinding and data collection

The providers and study investigators were aware of the treatment allocation, whereas the patients and laboratory technicians running the blood tests were blinded from the treatment. Randomization was performed by one of the investigators who was not involved in data collection. A sample size of 32 (16 in each arm) was calculated to provide 80% power to detect a difference of 0.10 IU/ml in the mean anti Xa levels between the two arms, with a standard deviation of 0.10. This is the hypothetical distance between the midway of the 0.2–0.4 target peak anti Xa range identified by expert opinion [15, 32]. Block randomization technique was utilized to allocate subjects into either arm. The Case Report Form (CRF) was specifically designed for this study, which collected information about patient demographics, comorbid illnesses, laboratory results, treatments, adverse events, and risk of developing VTE according to the PADUA score, among other information [34].

### Statistical methods

Data analysis was carried out using SPSS (version no.24). Characteristics of the study population were evaluated using descriptive statistics. Data was expressed as frequencies and percentages for categorical variables, means ± SD for numerical variables. Differences in proportions between the two study arms were evaluated using the Pearson chi-square or fisher exact test depending on cell size. Differences in means were tested using the t-test. Pearson correlation coefficient was used to estimate correlations between numerical variables. When the assumption for normality distribution was violated bootstrapping was used to correct for potential estimation bias. A two-sided *p* value < 0.05 was considered statistically significant.

## Results

**Fig. 1:**
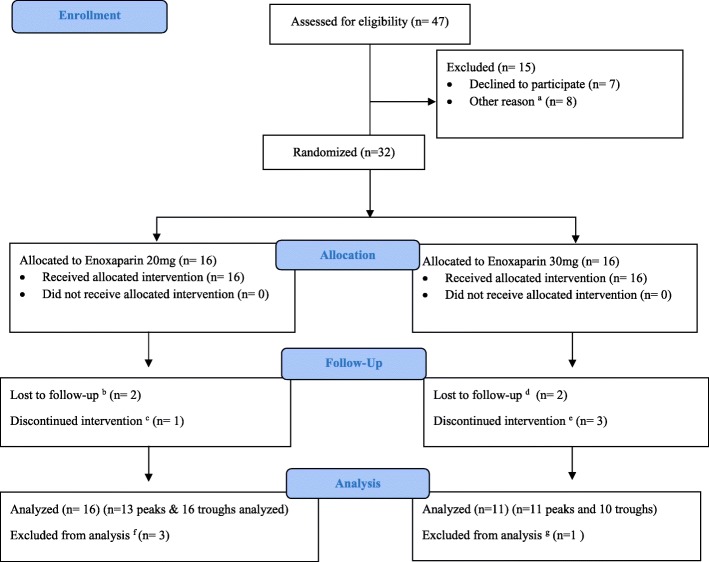
Flow chart. a Due to extreme body weight or due to primary physicians' preference to use other medications such as unfractionated heparin or tinzaparin. b Participant’s decision: peak anti-xa levels were not available in two patients because in one patient the blood was drawn incorrectly, and in the second patient participant refused to draw blood for peak level. c Investigator’s decision (Adverse event): participant experienced hematuria therefore treatment discontinued before the 3rd dose was given, however the trough level was drawn. d Discharged before levels were drawn. e Three patients discontinued the intervention due to investigator’s decision, one was a man who had low body weight 48 kg and elevated SrCr, second patient had their third dose discontinued before endoscopic biopsy, third patient was found to have a retroperitoneal bleed after one dose of enoxapain therefore the treatment was discontinued. f Primary outcome, peak anti-xa levels were not drawn in 3 patients as mentioned above in the follow up section. As such, only trough levels for these patients were available. g Trough anti-xa level was excluded because sample clotted

This study was conducted from October 2015 till July 2017. Forty-seven patients were assessed for eligibility. Thirty-two patients were randomized to 20 mg and 30 mg enoxaparin groups with 16 in each arm who received the allocated intervention (Fig. 1).

Table 1 presents patients’ baseline characteristics and studied parameters per treatment group. The mean length of follow up was 7 days in both groups. By day three of thromboprophylaxis, patients in the 20 mg arm had a statistically significant lower CrCl as compared to the 30 mg arm (Table 1). The most common VTE prophylaxis indications according to the PADUA score were reduced mobility, elderly age followed by acute infection, respiratory failure or heart failure and cancer. Eight patients in the study required intensive care unit (ICU) admission, (8/32) patients. In both groups, participants had a similar past medical history except for more patients with congestive heart failure (CHF) in the 20 mg enoxaparin group as compared to the 30 mg group (*p* = 0.02).

**Table 1:** Baseline characteristics

Characteristic^a^	Enoxaparin 20 mg SC Daily (*n* = 16)	Enoxaparin 30 mg SC Daily (*n* = 11)	*P*-value
Age (years)	83.8 ± 6.9	82.1 ± 6.4	0.52
BMI (Kg/m^2^)	24.1 ± 3.4	28.0 ± 6.3	0.16
Weight (Kg)	63.1 ± 8.3	70.2 ± 12.3	0.11
Padua score	3.5 ± 1.5	3.3 ± 2.1	0.74
Female Gender (%)	68.8 (11/16)	81.8 (9/11)	0.66
Alcohol Use (%)	18.8 (3/16)	36.4 (4/11)	0.39
ICU Admission (%)	37.5 (6/16)	18.2(2/11)	0.41
Chronic Kidney disease (%)	62.5 (10/16)	54.5 (6/11)	0.71
CrCl on day 1 (%)			
< 20	56.3 (9/16)	36.4 (4/11)	0.54
20–29	25.0 (4/16)	27.3 (3/11)	
30–35	18.8 (3/16)	36.4 (4/11)	
CAD (%)	43.8 (7/16)	45.5 (5/11)	0.99
Diabetes (%)	43.8 (7/16)	54.5 (6/11)	0.70
CHF (%)	56.3 (9/16)	9.1 (1/11)	0.02
Cancer (%)	25.0 (4/16)	36.4 (4/11)	0.68
Antiplatelet use on admission			
Aspirin (%)	56.3 (9/16)	54.5 (6/11)	0.99
Clopidogrel (%)	12.5 (2/16)	27.3 (1/11)	0.99
Concomitant medications during hospitalization			
ARBs (%)	25.0 (4/16)	9.1 (1/11)	0.51
Statin (%)	37.5 (6/16)	27.3 (3/11)	0.69
Aspirin 81 mg–100 mg (%)	56.2 (9/16)	63.6 (8/11)	0.99
Clopidogrel 75 mg (%)	18.7 (3/16)	9.1 (1/11)	0.62
Loop diuretics PO (%)	12.5 (2/16)	27.3 (3/11)	0.37
Loop diuretics IV (%)	50.0 (8/16)	36.4 (4/11)	0.70
Aldosterone antagonists (%)	6.2 (1/16)	9.1 (1/11)	0.99
Laboratory trends			
Lab day 1 potassium (mEq/L)	4.52 ± 0.93	4.60 ± 0.86	0.83
Lab day 3 potassium (mEq/L)	4.33 ± 0.77	3.95 ± 0.66	0.22
Lab day 1 hemoglobin (g/dL)	10.33 ± 2.08	11.29 ± 1.66	0.29
Lab day 3 hemoglobin (g/dL)	9.58 ± 1.51	10.48 ± 1.50	0.22
Lab day 1 Platelets, (per microliter)	270,692 ± 148,970	302,250 ± 133,511	0.63
Lab day 3 Platelets (per microliter)	206,333 ± 63,532	263,428 ± 86,061	0.12
Lab day 1 SCr (mg/dL)	2.62 ± 1.25	2.15 ± 1.13	0.34
Lab day 3 SCr (mg/dL)	2.82 ± 1.22	2.31 ± 1.76	0.37
Mean SCr (mg/dL)	2.72 ± 1.15	2.28 ± 1.46	0.39
Lab day 1 CrCl (mg/dL)	19.71 ± 8.76	24.57 ± 8.13	0.15
Lab Day 3 CrCl (mL/min)	17.52 ± 6.75	28.22 ± 14.52	0.02
Mean CrCl (mg/dL)	18.55 ± 7.33	25.97 ± 10.49	0.04

*Abbreviations*: *CrCl* creatinine clearance, *CAD* coronary artery disease, *CHF* congestive heart failure, *ARBs* angiotensin receptor blockers, *SCr* serum creatinine

^a^Data are mean ± SD values unless otherwise indicated

The assessment of anti-Xa levels was performed to compare the two treatment strategies and a statistically significant difference between both the mean peak and trough anti-Xa levels was noted between the arms. (Table 2) Seventy-three percent of patients in the enoxaparin 30 mg arm (8/11) achieved anti-xa levels within the recommended prophylactic peak range as compared to 38.50% (5/13) in the 20 mg arm, *p* = 0.09. In both arms, no patient experienced a peak anti-Xa level, above 0.5 IU/ml. Trough accumulation above 0.1 IU/ml was noted in one patient in each arm and there was no statistically significant difference between the arms (*p* = 0.99).

**Table 2:** Primary and Secondary outcomes: Peak and trough anti-xa levels achieved with each enoxaparin dose

Outcomes^a^	Enoxaparin 20 mg SC daily (*n* = 16)	Enoxaparin 30 mg SC daily (*n* = 11)	*P*-value
Peak anti-xa level (IU/mL)	0.14 ± 0.09 (0.08–0.19)	0.26 ± 0.11 (0.18–0.34)	0.004
Trough anti-xa level (IU/mL)	0.03 ± 0.04 (0.01–0.05)	0.06 ± 0.03 (0.04–0.08)	0.044

*Abbreviations*: *SC* Subcutaneous

^a^ Data are mean ± SD (95%CI) values unless otherwise indicated

**Fig. 2:**
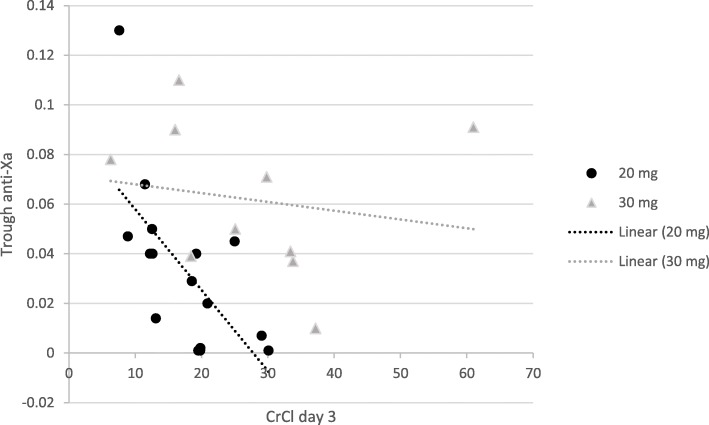
Correlation of Trough anti-Xa levels with creatinine clearance (CrCl) at day 3. For 20 mg group *r* = − 0.659 *p* = 0.005, for 30 mg *r* = − 0.173 *p* = 0.632

**Fig. 3:**
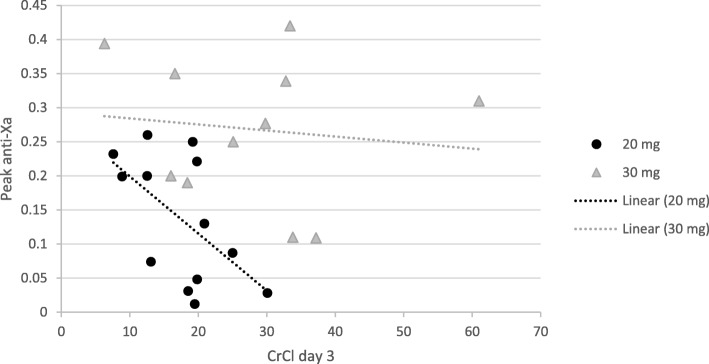
Correlation of Peak anti-xa levels with creatinine clearance (CrCl) at day 3. For 20 mg group *r* = − 0.570 *p* = 0.042, for 30 mg *r* = − 0.121 *p* = 0.724

Two patients experienced hemorrhage. One patient in the 20 mg arm, (1/16) experienced hematuria, a minor bleed, and one patient in the 30 mg arm, (1/16) experienced a retroperitoneal bleed and received multiple transfusions of packed RBCs, a major bleed. No VTE occurrences were noted in either arm. Thrombocytopenia and hyperkalemia occurred in one patient in the 20 mg arm. Both trough and peak anti-Xa levels were positively correlated with serum creatinine (SrCr) on day 3 in the 20 mg dose arm. Both levels were negatively correlated with CrCl on day 3 (Figs. 2 and 3).

## Discussion

To our knowledge, this is the first study measuring anti-Xa levels for enoxaparin 20 mg subcutaneously daily in elderly patients with renal impairment who are not on hemodialysis. This study showed that the mean activity anti-Xa levels, reflecting thromboprophylaxis efficacy, correlated with the subcutaneous dose of enoxaparin, with the 20 mg dose resulting in a mean anti-Xa activity level of half that of the 30 mg arm. This correlation between dose and anti-Xa levels is consistent with that reported in other studies [35–39]. In terms of safety, none of the patients exceeded the recommended anti-Xa levels, and there was no difference in bleeding events. Although patients in the 20 mg arm had a significantly lower CrCl as compared to the 30 mg arm, the former still had lower peak anti-Xa levels.

To our knowledge, there is no published data comparing neither anti-Xa nor clinical endpoints in elderly patients taking enoxaparin 20 mg compared to 30 mg subcutaneously daily for thromboprophylaxis in this patient population. In addition, amongst the studies assessing LMWH for thromboprophylaxis in patients with renal impairment, only very few studies have evaluated the incidence of VTE as the primary endpoint, while other studies evaluated anti-Xa levels or other pharmacodynamics and pharmacokinetic parameters [3, 5, 9, 17, 40]. There is limited literature suggesting that enoxaparin 20 mg subcutaneously is equally effective in elderly patients when compared to heparin 5000 units SC BID [41]. Recent literature supporting the use of enoxaparin 20 mg was limited by the study design that did not include a control group [26]. Data from the MEDENOX trial showed that 20 mg of enoxaparin was equivalent to placebo in reducing VTE events, but the trial excluded patients with a SrCr≥1.7 mg/dl or CrCl< 30 ml/min [6]. We acknowledge that the MEDENOX trial excluded patients with renal impairment, and that our cohort is composed of patients with renal impairment, however as evidenced by an anti-Xa levels published in subgroup analysis from the MEDENOX trial, enoxaparin 20 mg resulted in anti-Xa levels of 0.2 IU/ml [42]. In our study, even in patients with renal impairment, less than 50 % achieved anti-Xa levels of 0.2 IU/ml.

Peak anti-Xa levels were used as the primary endpoint since they have been shown to correlate more strongly with safety and efficacy than trough levels [43]. There is no clear consensus on peak anti-Xa levels for prophylactic doses of enoxaparin, however, many references recommend a level of < 0.5 IU anti-Xa /ml since anti-Xa target levels for VTE treatment dose range between 0.5–1.0 units/ml for twice daily regimens [12, 19, 44, 45]. Although anti-Xa levels as a surrogate marker has not been verified as an indicator of clinical antithrombotic efficacy in non-surgical patients, monitoring anti-Xa levels is recommended to guide dose optimization in high-risk patients [42, 46]. Furthermore, Levine et al. showed a statistically significant relationship between anti-Xa level and thrombosis among orthopedic patients receiving thromboprophylaxis [4, 47].

In our study, enoxaparin 20 mg yielded anti-Xa levels lower than those previously reported despite the fact that our peak levels were sampled on the third dose which could have allowed for accumulation. Sanderink et al. described a 29% increase in anti-Xa levels after 4 days of thromboprophylaxis in patients with a CrCl < 30 ml/min, which was explained by a prolonged half-life and decrease in renal elimination in comparison to healthier adults [5]. Although there is insufficient data to make assessments between anti-Xa levels and prophylactic efficacy, the lower than recommended mean anti-Xa levels observed in this trial should be taken into consideration pending the availability of clinical thrombotic endpoint data [9].

On day 3 of thromboprophylaxis, we found a positive and an inverse correlation between anti-Xa levels in the 20 mg arm with SrCr and CrCl, respectively. Such correlation may be due to the lower CrCl (mean of 17.3 ml/min) in the 20 mg arm, and this has been previously described in literature in patients with a CrCl< 30 ml/min [4].

Much of the published literature focuses on risk of bleeding in patients with renal dysfunction on thromboprophylaxis. In patients with advanced renal impairment (CrCl < 30 ml/min) receiving enoxaparin for VTE prophylaxis, the rates of bleeding reported in literature range between 0 and 6% [4, 9, 40]. Moreover, in patients with severe bleeding complications, the anti-Xa levels were either undetectable or the same as other patients in the study [4]. The rates of bleeding observed in our cohort were similar 6.25% (1/16) patients in each arm. Moreover, anti-xa levels do not appear to be related to bleeding risk in patients with significant renal impairment.

This trial represents the first study to characterize anti-Xa levels in patients on enoxaparin 20 mg versus 30 mg in elderly patients with renal impairment. This patient population is at an increased risk of bleeding and dose optimization or the selection of safer alternatives in renal impairment is essential to ensure a favorable risk/benefit ratio. We acknowledge the limitations to our study namely the small sample size, short duration of follow up and the use of a CrCl cut off of ≤35 ml/min instead of < 30 ml/min. This less stringent cut off was adopted based on the concern that CrCl may over predict the glomerular filtration rate [48]. Although dosing references recommend the CrCl as calculated by the Cockcroft-Gault formula for medication dose adjustments, clinical judgment is recommended based on the physiologic changes in elderly patients [49]. The lower lean body mass, reduced intake of proteins and malnutrition all affect the production and secretion of creatinine and therefore the CrCl. Moreover, in patients with acute kidney injury, the rise in serum creatinine usually lags behind the kidney injury, therefore not immediately reflecting the extent of the kidney injury [50]. Furthermore, two patients recruited into the study had received unfractionated heparin as thromboprophylaxis and were then switched to enoxaparin after 12 h. One patient in the 30 mg arm had received 1 dose of heparin 5000 SC BID, whereas one patient in the 20 mg arm had received 34 doses of heparin 5000 units SC BID. After excluding these patients from the analysis, the anti-Xa levels were still consistent with the results, enoxaparin 20 mg peak anti-Xa levels 0.13 ± 0.10 vs enoxaparin 30 mg 0.27 ± 0.11, *p* = 0.004 and enoxaparin 20 mg trough anti-xa levels were 0.03 ± 0.03 vs enoxaparin 30 mg 0.06 ± 0.03, *p* = 0.038. It is important to note that although CHF was more common in the 20 mg arm, which may have led to decreased subcutaneous absorption, a sensitivity analysis excluding all patients with CHF from both arms showed consistent findings with the overall study results. The enoxaparin 20 mg peak anti-Xa levels were 0.11 ± 0.10 vs enoxaparin 30 mg 0.26 ± 0.11, *p* = 0.010 and enoxaparin 20 mg trough anti-xa levels were 0.02 ± 0.02 vs enoxaparin 30 mg 0.06 ± 0.03, *p* = 0.012.

## Conclusion

In conclusion, this study showed that thromboprophylaxis with enoxaparin 30 mg provides higher control of anti-Xa activity in non-surgical elderly patients with renal impairment in comparison to enoxaparin 20 mg. In light of minimal available data evaluating the efficacy of enoxaparin 20 mg for thromboprophylaxis in renal impairment, and in context of the observed anti-xa levels, it may be safer for providers to use enoxaparin 30 mg SC.

## Abbreviations

CHF: Congestive heart failure
CrCl: Creatinine clearance
CRF: Case report form
DVT: Deep venous thrombosis
IBW: Ideal body weight
ICU: Intensive care unit
LMWHs: Low molecular weight heparins
PE: Pulmonary embolism
SC: Subcutaneously
SrCr: Serum creatinine
VTE: Venous thromboembolism
